# Retrospect of the Progress of Medicine and Surgery for the Year 1841-2

**Published:** 1843-01

**Authors:** 


Art. VII.—*Retrospect of the Progress of Medicine and Surgery for the year* 1841-2. By Mr. E. O. Spooner and Mr. W. Smart. *Read before the Southern Branch of the Provincial Medical Association.—Blandford,* 1842. 8vo, pp. 87.

This is another excellent retrospect of the same kind as the foregoing, and shows that the branches of the Provincial Association are worthy scions of the parent-stem. The fact of a work of such learning and research emanating from two general practitioners resident in small country towns, speaks well for the state of medical knowledge in the provinces. Thirty years ago such an event could scarcely have occurred; now, we believe, there are few districts in the kingdom which might not furnish a parallel to it.

